
JOURNAL INFORMATION
==============================
NLM Title Abbreviation: ScientificWorldJournal
Journal ID: ScientificWorldJournal
NLM Journal ID: 101131163
Journal ID: TSWJ

The Scientific World Journal

ISSN: 2356-6140
EISSN: 1537-744X
Publisher: Wiley

ARTICLE INFORMATION
==============================
PMCID: PMC6009730
PMID: 29973783
DOI: 10.1100/tsw.2002.9
Article ID: 658768
Article version: 1
Subjects: Short Report

Elucidation of Protein Structural and Pharmacophore Features Based on Sequence Clustering by Common Neighbor Comparisons

Kho Richard *
Hansen Mark
Baker Brian
Newman Joe
Sem Daniel S.
Jack Richard
Villar Hugo
Triad Therapeutics, Inc., 5820 Nancy Ridge Dr. Suite 200, San Diego, CA 92121, USA
*Richard Kho: rkho@triadt.com
Publication date: 2002
Electronic publication date: 2002 Jan 5
Volume: 2
First page: 17
Last page: 18
Copyright: Copyright © 2002 Richard Kho et al.
Copyright year: 2002
License: This is an open access article distributed under the Creative Commons Attribution License, which permits unrestricted use, distribution, and reproduction in any medium, provided the original work is properly cited.
License URL: https://creativecommons.org/licenses/by/3.0/

==============================
Advances in Gene Technology: The Genome and Beyond –
Structural Biology for Medicine (Proceedings of the 2002 Miami
*Nature Biotechnology* Winter Symposium)
*TheScientificWorld* 2002, 2(S2), 17–18
ISSN 1532-2246; DOI 10.1100/tsw.2002.9

### ELUCIDATION OF PROTEIN STRUCTURAL AND PHARMACOPHORE FEATURES BASED ON SEQUENCE CLUSTERING BY COMMON NEIGHBOR COMPARISONS

Richard Kho, Mark Hansen, Brian Baker, Joe Newman, Daniel S. Sem,
Richard Jack, and Hugo Villar
Triad Therapeutics, Inc., 5820 Nancy Ridge Dr. Suite 200, San Diego, CA 92121
*rkho@triadt.com

**INTRODUCTION.** Genome sequencing projects have dramatically increased our understanding of the pathways and proteins involved in biological systems. The NAD(P)-utilizing family of enzymes is one of the most important gene families representing 15% of all known enzyme functions. We have performed a complete characterization of this gene family on a sequence, structural, and NAD(P)-pharmacophore level. Genome-wide analysis was performed to gain a complete understanding of the structures and functional importance of NAD(P)-utilizing enzymes in 17 different genomes.

**METHOD.** The NAD(P)-utilizing enzymes were clustered on sequence, structure, and pharmacophore levels to identify biochemically meaningful relationships. Hidden Markov Models were built from the sequence clusters and used to characterize the NAD(P)-utilizing enzymes in proteomes[1].

**RESULTS.** A total of 92 NAD(P)-utilizing protein sequence families were identified in the Swiss-Protein database[2]. About 60% of the sequence families contained one or more structurally characterized members. In cases where there was more than one structure, the NAD(P)-binding domains were highly conserved. From these structures, 12 distinct NAD(P) conformational families were identified. Conformational family correlates with structure, which in turn strongly correlates with sequence. Shown in Fig. 1 is a subfamily of NAD(P)-utilizing enzymes representing 50 different catalytic functions, two sequence families, and one SCOP structural fold[3].

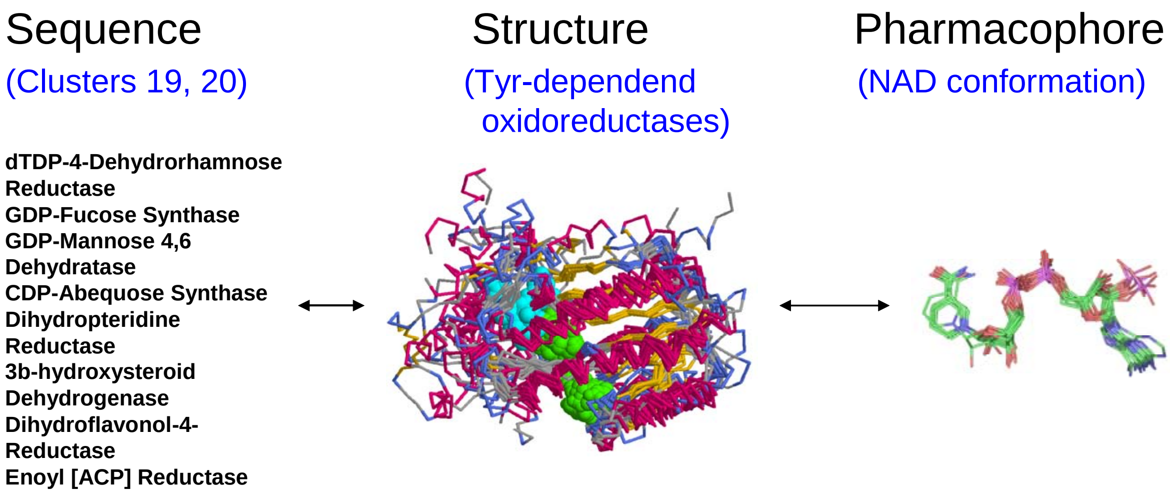

**Figure 1.** Sequence cluster, structure, pharmacophore correlation. Structural overlays of Tyrosine-dependent oxidoreductases show a conserved Rossmann fold

Genome-wide parsing based on sequence cluster profiles showed that approximately 2 to 5% of all ORFs in nonviral genomes belong to the NAD(P)-utilizing enzyme family. The two-domain Rossmann fold and short-chain oxidoreductases account for 73 to 95% of all NAD(P)-utilizing enzymes.

**DISCUSSION.** As shown in Fig. 1, a correlation can be made between sequence, structure, and NAD(P)-pharmacophore. The information gained can be used for a chemogenomics approach that leverages sequence information to reduce the effort required to identify ligands. In the case of NAD(P)-utilizing enzymes, it is likely to have a significant impact in the identification of novel anti-infective targets, among other possible areas.

### REFERENCES

1. Eddy, S.R. (1998) Bioinformatics 14, 755–763.
2. Bairoch, A. and Apweiler, R. (1998) Nucl. Acids Res. 26, 38–42.
3. Murzin A.G., Brenner S.E., Hubbard, T., and Chothia, C. (1995) J. Mol. Biol. 247, 536–540.
